# Biocompatibility of Dental Amalgams

**DOI:** 10.1155/2011/981595

**Published:** 2011-11-23

**Authors:** Yurdanur Uçar, William A. Brantley

**Affiliations:** ^1^Department of Prosthetic Dentistry, College of Dentistry, Cukurova University, Balcali, Saricam, 01330 Adana, Turkey; ^2^Division of Restorative and Prosthetic Dentistry, College of Dentistry, The Ohio State University, Columbus, OH 43210, USA

## Abstract

*Objective*. The purpose of this review paper is to review the literature regarding the toxicology of mercury from dental amalgam and evaluate current statements on dental amalgam. *Materials and Methods*. Two key-words “dental amalgam” and “toxicity” were used to search publications on dental amalgam biocompatibility published in peer-reviewed journals written in English. Manual search was also conducted. The most recent declarations and statements were evaluated using information available on the internet. Case reports were excluded from the study. *Results*. The literature show that mercury released from dental amalgam restorations does not contribute to systemic disease or systemic toxicological effects. No significant effects on the immune system have been demonstrated with the amounts of mercury released from dental amalgam restorations. Only very rarely have there been reported allergic reactions to mercury from amalgam restorations. No evidence supports a relationship between mercury released from dental amalgam and neurological diseases. Almost all of the declarations accessed by the internet stated by official organizations concluded that current data are not sufficient to relate various complaints and mercury release from dental amalgam. *Conclusions*. Available scientific data do not justify the discontinuation of amalgam use from dental practice or replacement with alternative restorative dental materials.

## 1. Introduction

The American Dental Association (ADA) defines dental amalgam as an alloy composed of mercury, silver, tin, and copper along with other metallic elements added to improve physical and mechanical properties [1]. Dental amalgam has been an accepted part of dental treatment for more than 170 years [2–5]. Mackert and Wahl (2004) reported that more than 75% of dentists in United States of America surveyed in 2001 placed dental amalgam restorations [6]. The ADA presented a recent estimate that more than 70 million dental amalgam restorations have been placed in the United States [7]. In 1999, about 60% of the restorations of Class I and II defects in the United States were restored with dental amalgam [8]. These percentages are even higher in developing countries.

Besides being prepared easily, dental amalgam is relatively inexpensive compared to most other materials used in dental treatment, and the longevity of dental amalgam restorations is relatively high [9]. Dental amalgam is easy to place in the prepared tooth, has low creep, high compressive strength and high resistance to wear, and experiences minimal dimensional change with time [1, 5]. It is the only dental material known for marginal-sealing capacity due to the corrosion products released from dental amalgam restorations [1, 4, 10]. It also tolerates a wide range of clinical placement conditions such as wet fields (for zinc-free products). However, toxicity of dental amalgam due to mercury has always been a concern. 

The purpose of this paper is to review the literature regarding the toxicology of mercury from dental amalgam and to evaluate current statements of different public agencies and councils on dental amalgam use.

## 2. Materials and Methods

Entering the two key words “dental amalgam” and “toxicity”, publications on dental amalgam biocompatibility published in peer-reviewed journals were searched in PubMed. A total of 379 papers were listed. When the search was limited to papers with abstracts and written in English, the number of papers was reduced to 198. Each abstract was read to evaluate whether the paper was relevant to the topic of the current review paper. Using the online library of Çukurova University, 43 out of 98 manuscripts that were relevant to the topic of the current review paper were accessed. A manual search was also conducted to find additional articles related to the biocompatibility of dental amalgam. The most recent declarations and statements from public agencies and councils were also evaluated. Case reports were excluded from this study.

## 3. Toxicology of Mercury from Dental Amalgam

Even if dental amalgam has provided excellent clinical service for many years and there are only extremely rare cases of documented adverse effects [9], dental amalgam has always generated some concerns [11, 12] due to the mercury (Hg) content which is around 40–55% [13]. Mercury, which is the only metal in the liquid phase at normal room temperature, has a high vapor pressure that increases rapidly with temperature [14]. (The other metal that is in the liquid state near room temperature is gallium, which has a melting temperature near 30°C.)

People can be exposed to mercury from diet, drinking water, air, and dental amalgam restorations. Dental amalgam is prepared by mixing the alloy for dental amalgam powder with mercury. Mercury is released from dental amalgam mainly in the form of elemental mercury vapor. Mercury vapor in humans has been sampled in exhaled breath [15], in the oral cavity [16, 17] with the mouth open or closed, and through a catheter placed in the trachea via a bronchoscope [18]. The data from these studies suggest that mercury is continuously released in the oral cavity from dental amalgam restorations. The release rate is dependent upon many factors including area, age, eating and individual habits, composition of the amalgam, and the quantity of the surface oxide layer. Mercury vapor dissolves in the intraoral air or saliva. Then, it enters the organism via different routes. Exposure to mercury from dental amalgam restorations occurs through several ways: (1) mouth air containing elemental mercury released from the dental amalgam can be inhaled; (2) dental amalgam particles can be abraded from restored surfaces during mechanical wear of the restorations or can be produced during placement or replacement of the restorations, and abraded particles from the restorations can be ingested; (3) the saliva into which both elemental and corrosion-produced inorganic mercury products are dissolved can be swallowed; (4) “tattooing” may be created when particles from the restorations are physically embedded in soft tissue adjacent to the restoration area.

Lorscheider and his coworkers (1995) pointed out that dental amalgam restorations were the major contributing source of mercury in humans who were not occupationally exposed to mercury and reported that research evidence had not supported the safety of dental amalgam at that time [11]. Mercury vapor can be released from dental amalgam during all steps involved with the restorations like trituration, condensation, setting, polishing, and removal. Mastication and drinking hot beverages cause release of mercury from dental amalgam restorations as well [16]. However, the World Health Organization (WHO) has announced that eating seafood once in a week raises urine mercury level to 5–20 *μ*g/L, which is higher than the exposure from dental amalgam (1 *μ*g/L) [19]. On the other hand, the amount of mercury vapor that is accepted by the Occupational Safety and Health Administration (OSHA) in the United States is 100 times more than the amount to which a person with 9 dental amalgam restorations will be exposed [19]. The maximum amount of mercury vapor allowed in the workplace, the Threshold Limit Value (TLV), is set as 0.05 mg/m^3^ by OSHA. In their textbook on restorative dental materials, Craig and Powers reported (2002) that fetuses exposed to mercury concentrations of 5 mg/m^3^, which is far beyond the TLV, were stillborn [19].

Abrasive stress (like chewing and brushing) on exposed surfaces of dental amalgam restorations can alter the protective characteristics of the oxide layer formed at the surface and increase the elemental mercury release rate [15]. Dental amalgam restorations release not only elemental mercury, but also inorganic mercury, as corrosion products [4]. Almost all of the elemental mercury is converted to inorganic mercury. The clinical use of dental amalgam for restorations can also alter the mercury-containing phases, *γ*_1_ and *γ*_2_, and cause mercury release; most of the released mercury reacts with unreacted particles of the starting alloy for dental amalgam and only little of it escapes from the restoration [20].

Medical research has demonstrated that mercury is continuously released as vapor into the air, then inhaled, absorbed into body tissues, oxidized to ionic Hg, and finally covalently bound to cell proteins [11]. Unlike some other groups reporting the highest accumulation levels of mercury in the kidneys [21, 22], for cases with more than 12 amalgam surfaces Guzzi and his coworkers (2006) reported the highest level of mercury accumulation in the brain of 18 cadavers compared to the thyroid and renal cortex [12]. Accumulation of mercury in other organs like the lungs, liver, gastrointestinal tract, and exocrine glands has also been reported [23]. Furthermore, long-term dermal exposure to inorganic mercury may also lead to toxicity. 

It was reported that mercury levels in the kidneys, thyroid, and brain were higher in cadavers with higher numbers of amalgam surfaces [12]. The highest mercury concentration was found in the cerebral cortex and the pituitary gland. Barregard et al. (2010) reported that dental amalgam was the main source of mercury in the kidneys [24]. Increased mercury levels in the liver, spleen, and lungs with increased numbers of amalgam restorations were also reported [23]. Mercury concentrations were reported to be 2-3 fold and 9-fold higher, respectively, in the brain and kidneys of people with dental amalgam restorations compared with those without these restorations.

Okabe et al. (2003) reported greater release rate of mercury from high-copper dental amalgams with single-composition starting alloy particles that are mixed with mercury, compared to dental amalgams prepared from an admixture of low-copper and high-copper starting particles [25]. It should be noted that an increase in mercury level does not mean that the biochemical function of the previously mentioned organs would have been changed. Dunsche et al. (2003) reported a relationship between oral lichenoid reactions and dental amalgam restorations and that 97.1% of patients benefited from removal of these restorations [26].

It has been reported that elemental mercury, which has a limited ability to cross biological membrane, can nonetheless cross the placenta and the blood-brain barrier after being dissolved in blood and then become distributed throughout the body. This can be attributed to the high lipophilicity of elemental mercury [27] that is the origin of mercury retention in the brain and fetal tissues if an overdose is taken [18]. A prospective, blinded epidemiological study was carried out to evaluate the relationship between mercury exposures from maternal dental amalgam restorations during pregnancy [28]. This group reported an increased risk of autism severity at the threshold of 6 or more maternal dental amalgam restorations during pregnancy and early infant temporal periods. However, Lindbohm et al. (2007) investigated whether dental workers exposed to acrylate compounds, dental amalgam, solvents, or disinfectants are at an increased risk of miscarriage [29]. They did not find a strong association or a consistent dose-response relationship between exposure to chemical agents in the dental workplace and the risk of miscarriage. 

The New England Children's Amalgam Trial was completed on 534 children aged 6 to 10 years at baseline [30]. The neuropsychological and renal functions of children whose dental caries were restored using dental amalgam (*n* = 267) or resin composite (*n* = 267) were compared. A 5-year-followup study was conducted between September 1997 and March 2005. The 5-year change in full-scale IQ scores and tests of memory and visuomotor ability was evaluated. Renal glomerular function was measured by creatinine-adjusted albumin in urine. The authors reported no statistically significant differences in adverse neuropsychological or renal effects observed in children whose caries were restored using dental amalgam or composite resins. A followup study of The New England Children's Amalgam Trial was completed to compare full-scale IQ score, general memory index, and visual motor abilities [31]. The authors reported that dental amalgam was not associated with an increase in the risk of children experiencing neuropsychological dysfunction. 

Another randomized clinical trial was performed to assess the safety of dental amalgam restorations in 507 children in Lisbon, Portugal [32]. Children were randomized to either dental amalgam (*n* = 253) or resin composite (*n* = 254) restorations, and only children aged 8 to 10 years with at least 1 carious lesion on a permanent tooth were included in the study. Memory, attention/concentration, motor/visuomotor domains, and nerve conduction velocities were measured. A statistically significant difference in neurobehavioral assessments or in nerve conduction velocity was not found among the two treatment groups. These authors suggested that dental amalgam should remain as a viable restorative option for children.

A further randomized, prospective trial examining the safety of dental amalgam was conducted (*N* = 507) on children aged between 8 through 12 years [33]. Annual clinical neurological examinations were conducted. The authors concluded that exposure to mercury from dental amalgam did not adversely affect neurological status in children.

Unlike the reports from the aforementioned studies, a JAMA Editorial Needleman (2006) presented an opposing view on the neurotoxic effects of dental amalgam [34]. He urged further examination of the molecular effects of dental amalgam at appropriate doses, with consideration of the exposure as precisely as possible, along with the vulnerability factors. 

The reported values of mercury release from dental amalgam restorations are controversial. One worst case estimate of Hg loss was 2 *μ*g/day [35] while another article reported a lesser amount [36]. However, one group has reported an Hg release up to 20–25 *μ*g/day from dental amalgam restorations [12]. The lowest dose of mercury that can start a toxic reaction is reported as 3–7 *μ*g/kg body weight [19]. Same authors reported that 500 *μ*g Hg/kg of body weight causes paresthesia, while 1000 *μ*g Hg/kg of body weight causes ataxia. Still higher doses of 2000 *μ*g Hg/kg and 4000 *μ*g Hg/kg of body weight can cause joint pain and hearing loss, respectively [19]. It should be noted that these doses are enormously greater than the mercury values that can be released from dental amalgam. 

The body cannot retain metallic mercury [19]. Metallic mercury will be disposed through urine, and urine mercury levels can be used for determining exposure to inorganic mercury [37]. Mercury levels in urine caused by dental amalgam restorations can be monitored by using radioactive mercury in the dental amalgam. Concentrations from 1–5 mg Hg/L urine are considered to be within the normal range. Symptoms of mercury poisoning have been reported at concentrations above 25–50 mg Hg/L urine. Neurological changes can be observed only when the urine mercury level is higher than 500 *μ*g/L, and it should be noted that this level is nearly 170 times the peak levels that have been found when a dental amalgam restoration is placed. Whether given elevated mercury level in urine is high enough to be harmful for the body should also be questioned. Urinary mercury levels in children were reported to be highly correlated with both number of dental amalgam restorations and time since placement [38]. Olstad et al. (1987) and coworkers reported a significant positive correlation between an increased number of surfaces of dental amalgam restorations and urine mercury [39]. However, this group stated that a consensus should not be driven by the foregoing correlation, since the observed levels of urine mercury were below any value of toxicological significance [40]. They reported that there was no correlation between urine Hg and allergy, as well as between the extent of dental amalgam restorations and allergy. 

Although mercury vapor is released from amalgam restorations, research over the past decades has failed to identify deleterious health outcomes. This can be attributed to insufficient mercury being released from dental amalgam restorations to cause a medical problem. Although Pesch et al. (2002) reported that mercury analysis in urine was a suitable way to estimate mercury exposure due to dental amalgam restorations [41], there are studies arguing that mercury in urine will not represent the mercury toxicity and/or mercury content in other body fluids or tissues [42, 43] and hence should not be used to measure mercury toxification. The latter group stated that the mercury concentration in critical organs of humans can be neither directly measured nor reliably estimated by means of media such as blood or urine [43]. Nevertheless, the increased amount of mercury in urine might imply that body can metabolize mercury to some extent and that the reported increased levels of mercury in urine fail to pose a risk of mercury toxicity from dental amalgam restorations [44]. On the other hand, Woods et al. (2007) reported significantly higher concentrations of urine mercury in girls compared to boys, which might suggest a possible sex-related difference in susceptibility to mercury toxicity [38].

Barregard et al. (2008) evaluated the renal effects of dental amalgam restorations for children [45]. No significant differences between treatment groups for the average levels of renal biomarkers (urinary excretion of albumin, alpha-1-microglobulin, *γ*-glutamyl transpeptidase, and *N-*acetyl*-β-*D-glucosaminidase) were found. This group reported that the number of dental amalgams that yielded these biomarkers was not significant, either. The only significant difference between dental amalgam and resin composite restoration groups was the increased prevalence of microalbuminuria (MA) among children in the dental amalgam group in years 3–5, which was stated as a possible random finding.

Mercury exposure from dental amalgam restorations has been calculated by measuring the total blood mercury level using atomic absorption spectroscopy [46]. The maximum medically acceptable level of mercury in the blood is 3 *μ*g/L [19]. Skoner et al. (1996) reported toxic and lethal doses of mercury in blood as 200 ng/mL and 600 ng/mL, respectively [47]. There are controversial results on blood mercury concentrations. Melchart et al. (2008) reported the amount of inorganic mercury in erythrocytes and plasma as 0.37 ng/mL and 0.38 ng/mL, respectively [43], and this group reported the total plasma mercury level as 0.49 ng/mL. Nur Özdabak et al. (2008) reported that dental amalgam restorations were the major source of plasma mercury (3.91 ng/mL), yet dental amalgam was not found to have a significant effect on plasma-total antioxidant activities [48]. The foregoing controversial results can be attributed to different methods used for evaluating the total plasma mercury concentrations and the influence of other mercury sources like diet, drinking water, and inhaled air, which might increase the blood mercury concentration. It is not possible to differentiate the amount of mercury taken from different sources. It is also very difficult to directly associate a mercury increase in blood to the mercury released from dental amalgam restorations. Blood mercury levels will increase to 1-2 *μ*g/L during placement of these restorations, and there is a decrease in blood mercury level after removal of dental amalgam restorations. 

The consequences of mercury release from dental amalgam, its absorption, accumulation, and excretion by the body, along with the ill effects of cumulative storage have been reviewed [49, 50]. These earlier reviews concluded that the low levels of mercury in body fluids reported in the literature were not likely to constitute a health hazard. The mercury release rate from dental amalgam restorations has also been investigated using saliva and breath mercury levels as biomarkers [51]. Using an in vitro model, they measured the air mercury levels for dry and saliva-coated dental amalgam discs and found higher air mercury levels for dry dental amalgam compared to wet dental amalgam. The release of mercury was higher from abraded dental amalgam compared to fresh dental amalgam. 

When mercury is mixed with the alloy particles for dental amalgam, there is a setting chemical reaction [20]. However, the amount of liquid mercury mixed with these alloy particles is insufficient to consume the starting alloy powder particles completely [19]. Therefore, the set amalgam contains incompletely consumed dental amalgam alloy powder particles (historically termed “core”), along with the reaction phases (historically termed “matrix”). Research has shown that the *γ*_1_ (Ag_2_Hg_3_) phase contains a small amount of tin and that it transforms to the *β* phase over long periods of time. The mercury released from the *γ*_2_ (Sn_8_Hg) phase [52] will further react with the unreacted alloy particles, and only minute amounts of Hg vapor can be released from set amalgam [20]. The amount of mercury released from dental amalgam restorations has been overestimated [16, 17].

Even if they are rare, allergic reactions to mercury do occur for patients with dental amalgam restorations [19, 53, 54]. There are case reports of allergic contact dermatitis, gingivitis, stomatitis, and remote cutaneous reaction to dental amalgam restorations. Allergic reactions to dental amalgam usually disappear in a couple of days or after removal of these restorations [19].

Release of corrosion products is another important issue about dental amalgam. Atomic emission spectroscopy and atomic absorption spectroscopy have been used to measure the mercury release from dental amalgam into various media [12]. The ionic dissolution will be very low once the dental amalgam is set. Low-copper dental amalgams release more ions than high copper ones because they are more prone to corrosion. Likewise, unpolished specimens will release greater amounts of mercury and silver [12]. 

Neurotoxicological effects of dental amalgam have also been investigated. Ritchie et al. (2002) reported that although several differences in health and cognitive functioning were found between dentists and control subjects, these differences could not be directly attributed to exposure to mercury [55]. Another group investigated the associations between Hg and symptoms, mood, motor function, and nonspecific cognitive alterations in task performance and reported that symptoms were similar in an occupationally exposed group to Hg and the general US population [56]. Sweeney et al. (2002) declared that amalgam placement appeared to present minimal mercury exposure risk from a neurotoxicological point of view [51]. 

Jones (1999) reported that there is no conclusive evidence in the scientific literature to demonstrate a link between the causes of irreversible neurological disorders or impaired kidney function and mercury vapor from dental amalgam restorations [57]. Animal experiments to date have not been able to establish any conclusive cause-and-effect link that can be extrapolated to human exposure to mercury from dental amalgam restorations [57–59]. Mercury pollution from dentistry is considered to be insignificant compared to that from industrial use and natural sources [60].

## 4. Statements from Different Agencies on Dental Amalgam Use

The safety of dental amalgam for restorative treatment has been reviewed many times by different agencies in the United States. The US Public Health Service (USPHS) published a broad scientific report about the safety of dental amalgam in 1993 [18], and the conclusions of this report were reaffirmed in 1995 and 1997 [61, 62]. The USPHS analyzed 175 peer-reviewed studies and reported that the data in these studies did not warrant a conclusion that mercury release from dental amalgam restorations will cause neurologic, renal, or developmental problems. On the other hand, previous studies have documented that dental amalgam restorations can cause allergic or hypersensitivity reactions although they are rare.

Even if most agencies agree that the available data do not confirm a health hazard caused by dental amalgam restorations, there are some countries that restrict or limit the use of dental amalgam. Health Canada (1996) has recommended that the use of dental amalgam is to be avoided for hypersensitive individuals or people with impaired kidney function, children, and pregnant women [63]. The German Ministry of Health (1997) and the Commission of the European Union (2008) have also stated that dental amalgam restorations should not be placed for these groups of people who are hypersensitive [64, 65], have impaired function, or lie in other special categories (LSRO, 2004) [54]. Recently, the European Commission (2008) reported no clinical justification to remove clinically satisfactory dental amalgam restorations. Those patients who are suspected to have allergic reactions and positive patch tests should be excluded [65].

The Council of Scientific Affairs of the American Dental Association (ADA) concluded in 1998 that amalgam continues to be a safe and effective restorative material in view of scientific information available at that time [44], and the ADA affirmed this statement in 2002, 2003, and 2009 [1, 7, 66]. The ADA stated that if the organization considered that dental amalgam posed a threat to the health of dental patients, they would advise their members to cease using this material for restorations. The ADA has concluded that dental amalgam offers a safe and cost-effective treatment option. Recently, the Council of European Dentists (CED) declared that dental amalgam continued to be the most appropriate material for many restorations due to its ease of use, durability, and cost-effectiveness (CED, 2010) [67].

The US Food and Drug Administration (FDA) published its statement on dental amalgam in December 2002 [53]. It was reported that this organization continues to investigate the safety of dental amalgam and that there is presently no valid scientific evidence which has shown that dental amalgam restorations causes harm to patients.

The National Institute of Dental and Craniofacial Research (NIDCR) of the US National Institutes of Health funded a project that had been performed by the Life Sciences Research Office (LSRO) [54]. The LSRO was asked to examine the peer-reviewed, primary scientific, and medical literature published between January 1, 1996 and December 31, 2003 relating to dental amalgam and human health. Approximately 300 studies out of 950 met the scientific and study design criteria and were used to construct the final report. The review was mainly based on the studies of mercury vapor or dental amalgam exposure in humans. The report concluded that there was little evidence to support a causal relationship between mercury in dental amalgam restorations and health problems for patients. The report also noted that there were existing research gaps, which, if addressed, may settle the dental amalgam controversy. For more detailed information on review and analysis of the literature on the potential adverse health effects of dental amalgam, the LSRO website can be accessed at http://www.lsro.org.

## 5. Conclusions

According to the available articles and data reviewed in this paper, the following conclusions can be drawn.

1. Mercury released from dental amalgam restorations does not contribute to systemic disease or systemic toxicological effects.
2. Allergic reactions to mercury from dental amalgam restorations have been demonstrated, but these are extremely rare.
3. Available scientific data do not justify the discontinuation of dental amalgam use from clinical practice or the replacement with other single-tooth restorative dental materials. There are cases where dental amalgam is the only choice with no other alternative.

## References

[1] American Dental Association (ADA) Council on Scientific Affairs Statement on dental amalgam. http://www.ada.org/1741.aspx.

[2] Greener EH (1979). Amalgam—yesterday, today, and tomorrow. *Operative Dentistry*.

[3] Eggleston DW (1989). Dental amalgam: a review of the literature. *Compendium*.

[4] Anusavice KJ (2003). *Phillips’ Science of Dental Materials*.

[5] George GN, Singh SP, Hoover J, Pickering IJ (2009). The chemical forms of mercury in aged and fresh dental amalgam surfaces. *Chemical Research in Toxicology*.

[6] Mackert JR, Wahl MJ (2004). Are there acceptable alternatives to amalgam?. *Journal of the California Dental Association*.

[7] American Dental Association Survey Center (2002). *The 1999 Survey of Dental Services Rendered*.

[8] Berthold M (2002). Restoratives: trend data shows shift in use of materials. *ADA News*.

[9] Roulet JF (1997). Benefits and disadvantages of tooth-coloured alternatives to amalgam. *Journal of Dentistry*.

[10] Swartz ML, Phillips RW (1961). *In vitro* studies on the marginal leakage of restorative materials. *Journal of the American Dental Association*.

[11] Lorscheider FL, Vimy MJ, Summers AO (1995). Mercury exposure from “silver” tooth firings: emerging evidence questions a traditional dental paradigm. *The FASEB Journal*.

[12] Guzzi G, Grandi M, Cattaneo C (2006). Dental amalgam and mercury levels in autopsy tissues: food for thought. *The American Journal of Forensic Medicine and Pathology*.

[13] Sarkar NK, Park JR (1988). Mechanism of improved corrosion resistance of Zn-containing dental amalgams. *Journal of Dental Research*.

[14] Okabe T (1987). Mercury in the structure of dental amalgam. *Dental Materials*.

[15] Patterson JE, Weissberg BG, Dennison PJ (1985). Mercury in human breath from dental amalgams. *Bulletin of Environmental Contamination and Toxicology*.

[16] Vimy MJ, Lorscheider FL (1985). Serial measurements of intra-oral air mercury: estimation of daily dose from dental amalgam. *Journal of Dental Research*.

[17] Vimy MJ, Lorscheider FL (1985). Intra-oral air mercury released from dental amalgam. *Journal of Dental Research*.

[18] Department of Health and Human Services Public Health Service Dental amalgam: a scientific review and recommended Public Health Service strategy for research, education and regulation: final report of the subcommittee on risk management of the Committee to Coordinate Environmental Health and Related Programs. http://web.health.gov/environment/amalgam1/ct.htm.

[19] Craig RG, Powers JM (2002). *Restorative Dental Materials*.

[20] Okabe T, Butts MB, Mitchell RJ (1983). Changes in the microstructures of silver-tin and admixed high-copper amalgams during creep. *Journal of Dental Research*.

[21] Clarkson TW (1997). The toxicology of mercury. *Critical Reviews in Clinical Laboratory Sciences*.

[22] Hussain S, Atkinson A, Thompson SJ, Khan AT (1999). Accumulation of mercury and its effect on antioxidant enzymes in brain, liver, and kidneys of mice. *Journal of Environmental Science and Health B*.

[23] Enwonwu CO (1987). Potential health hazard of use of mercury in dentistry: critical review of the literature. *Environmental Research*.

[24] Barregard L, Fabricius-Lagging E, Lundh T (2010). Cadmium, mercury, and lead in kidney cortex of living kidney donors: impact of different exposure sources. *Environmental Research*.

[25] Okabe T, Elvebak B, Carrasco L, Ferracane JL, Keanini RG, Nakajima H (2003). Mercury release from dental amalgams into continuously replenished liquids. *Dental Materials*.

[26] Dunsche A, Kästel I, Terheyden H, Springer ING, Christophers E, Brasch J (2003). Oral lichenoid reactions associated with amalgam: improvement after amalgam removal. *British Journal of Dermatology*.

[27] World Health Organisation (WHO) (2003). *Elemental Mercury and Inorganic Mercury Compounds: Human Health Aspects*.

[28] Geier DA, Kern JK, Geier MR (2009). A prospective study of prenatal mercury exposure from maternal dental amalgams and autism severity. *Acta Neurobiologiae Experimentalis*.

[29] Lindbohm M-L, Ylöstalo P, Sallmén M (2007). Occupational exposure in dentistry and miscarriage. *Occupational and Environmental Medicine*.

[30] Bellinger DC, Trachtenberg F, Barregard L (2006). Neuropsychological and renal effects of dental amalgam in children: a randomized clinical trial. *Journal of the American Medical Association*.

[31] Bellinger DC, Trachtenberg F, Daniel D, Zhang A, Tavares MA, McKinlay S (2007). A dose-effect analysis of children’s exposure to dental amalgam and neuropsychological function the New England children’s amalgam trial. *Journal of the American Dental Association*.

[32] DeRouen TA, Martin MD, Leroux BG (2006). Neurobehavioral effects of dental amalgam in children: a randomized clinical trial. *Journal of the American Medical Association*.

[33] Lauterbach M, Martins IP, Castro-Caldas A (2008). Neurological outcomes in children with and without amalgam-related mercury exposure: seven years of longitudinal observations in a randomized trial. *Journal of the American Dental Association*.

[34] Needleman HL (2006). Mercury in dental amalgam—a neurotoxic risk?. *Journal of the American Medical Association*.

[35] Marshall SJ, Marshall GW (1992). Dental amalgam: the materials. *Advances in Dental Research*.

[36] Mackert JR (1991). Dental amalgam and mercury. *The Journal of the American Dental Association*.

[37] Hörsted-Bindslev P (2004). Amalgam toxicity—environmental and occupational hazards. *Journal of Dentistry*.

[38] Woods JS, Martin MD, Leroux BG (2007). The contribution of dental amalgam to urinary mercury excretion in children. *Environmental Health Perspectives*.

[39] Olstad ML, Holland RI, Wandel N, Pettersen AH (1987). Correlation between amalgam restorations and mercury concentrations in urine. *Journal of Dental Research*.

[40] Olstad ML, Holland RI, Pettersen AH (1990). Effect of placment of amalgam restorations on urinary mercury concentration. *Journal of Dental Research*.

[41] Pesch A, Wilhelm M, Rostek U (2002). Mercury concentrations in urine, scalp hair, and saliva in children from Germany. *Journal of Exposure Analysis and Environmental Epidemiology*.

[42] Hultman P, Lindh U, Hörsted-Bindslev P (1998). Activation of the immune system and systemic immune-complex deposits in Brown Norway rats with dental amalgam restorations. *Journal of Dental Research*.

[43] Melchart D, Köhler W, Linde K (2008). Biomonitoring of mercury in patients with complaints attributed to dental amalgam, healthy amalgam bearers, and amalgam-free subjects: a diagnostic study. *Clinical Toxicology*.

[44] American Dental Association (ADA) Council on Scientific Affairs (1998). Dental amalgam: update on safety concerns. *The Journal of the American Dental Association*.

[45] Barregard L, Trachtenberg F, McKinlay S (2008). Renal effects of dental amalgam in children: the New England children’s amalgam trial. *Environmental Health Perspectives*.

[46] Eyeson J, House I, Yang YH, Warnakulasuriya KA (2010). Relationship between mercury levels in blood and urine and complaints of chronic mercury toxicity from amalgam restorations. *British Dental Journal*.

[47] Skoner JR, Wallace JA, Fochtman F, Moore PA, Zullo T, Hoffman RD (1996). Blood mercury levels with amalgam retroseals: a longitudinal study. *Journal of Endodontics*.

[48] Nur Özdabak H, Karaoğlanoğlu S, Akgül N, Polat F, Seven N (2008). The effects of amalgam restorations on plasma mercury levels and total antioxidant activity. *Archives of Oral Biology*.

[49] Eley BM, Cox SW (1993). The release, absorption and possible health effects of mercury from dental amalgam: a review of recent findings. *British Dental Journal*.

[50] Mackert JR, Berglund A (1997). Mercury exposure from dental amalgam fillings: absorbed dose and the potential for adverse health effects. *Critical Reviews in Oral Biology and Medicine*.

[51] Sweeney M, Creanor SL, Smith RA, Foye RH (2002). The release of mercury from dental amalgam and potential neurotoxicological effects. *Journal of Dentistry*.

[52] Mahler DB, Adey JD, Van Eysden J (1975). Quantitative microprobe analysis of amalgam. *Journal of Dental Research*.

[53] United States Food and Drug Administration Regulation of dental amalgams. http://www.fda.gov/NewsEvents/Testimony/ucm115161.htm.

[54] Life Sciences Research Organization (LSRO) (2004). *Report on Health Effects of Mercury from Dental Amalgam*.

[55] Ritchie KA, Gilmour WH, Macdonald EB (2002). Health and neuropsychological functioning of dentists exposed to mercury. *Occupational and Environmental Medicine*.

[56] Echeverria D, Aposhian HV, Woods JS (1998). Neurobehavioral effects from exposure to dental amalgam Hg°: new distinctions between recent exposure and Hg body burden. *The FASEB Journal*.

[57] Jones DW (1999). Exposure or absorption and the crucial question of limits for mercury. *Journal of the Canadian Dental Association*.

[58] Jones DW (2004). Putting dental mercury pollution into perspective. *British Dental Journal*.

[59] Jones DW (2008). Has dental amalgam been torpedoed and sunk?. *Journal of Dental Research*.

[60] Jones DW (2008). A Scandinavian tragedy. *British Dental Journal*.

[61] United States Public Health Service

[62] U.S. Department of Health and Human Services Working Group on Dental Amalgam Dental amalgam and alternative restorative materials an update report to the Environmental Health Policy Committee. http://web.health.gov/environment/amalgam2/Contents.html.

[63] Health Canada The safety of dental amalgam. http://www.hc-sc.gc.ca/dhp-mps/md-im/applic-demande/pubs/dent_amalgam-eng.php.

[64] German Ministry of Health

[65] European Commission. SCENIHR (Scientific Committee on Emerging and Newly-Identified Health Risks) Scientific opinion on the safety of dental amalgam and alternative dental restoration materials for patients and users. http://ec.europa.eu/health/ph_risk/committees/04_scenihr/docs/scenihr_o_016.pdf.

[66] ADA Council on Scientific Affairs (2003). Direct and indirect restorative materials. *The Journal of the American Dental Association*.

[67] Council of European Dentists. CED resolution Dental amalgam. http://www.eudental.eu/index.php?referer_ID=3733&ID=2741&marker=CouncilofEuropeanDentists.

